# Expansion resistance against genotoxic *E. coli*: protective effects of *Bifidobacterium pseudolongum* in DSS-induced colitis in mice

**DOI:** 10.3389/fmicb.2026.1877186

**Published:** 2026-07-28

**Authors:** Ayodeji Samuel Ajayi, Gabriela Fragoso, Annie Calvé, Claire Gerkins, Thibault Maumy, Claire McCartney, Manuela M. Santos

**Affiliations:** 1 Nutrition and Microbiome Laboratory, Institut du cancer de Montréal, Centre de recherche du Centre hospitalier de l'Université de Montréal (CRCHUM), Montréal, QC, Canada; 2 Department of Medicine, Faculty of Medicine, Université de Montréal, Montréal, QC, Canada

**Keywords:** acetate, *Bifidobacterium pseudolongum*, colibactin, colitis, gut microbiota, pathobiont, *pks*+ *E. coli*, putrescine

## Abstract

**Introduction:**

Ulcerative colitis is a chronic inflammatory disease affecting the colon, and its incidence is rising worldwide. Significant alterations to the composition and function of the gut microbiota that favor the expansion of pathobionts have been shown to be involved in ulcerative colitis. One such pathobiont is *Escherichia coli* strains carrying the *pks* island (*pks*+ *E. coli*) that contribute to mucosal injury and exacerbate inflammation. In this study, we investigated whether microbiota-targeting interventions may restrict *pks*+ *E. coli* expansion and mitigate chemically induced colitis in mice.

**Results:**

In mice colonized with the murine *pks*+ *E. coli* NC101 strain (EcNC101) in which acute colitis was induced by dextran sulfate sodium, oral administration of putrescine significantly reduced disease severity, histological damage, and inflammatory cytokine expression. 16S rRNA gene sequencing revealed marked shifts in gut microbiota composition following putrescine treatment, characterized by an increase in the relative abundance of Actinobacteriota and a decrease of Proteobacteria phyla. Among these, *Bifidobacterium pseudolongum* was associated with higher colonic acetate levels and reduced colitis severity. In EcNC101-colonized mice with colitis, *B. pseudolongum* administration significantly alleviated colitis by increasing acetate levels and activating peroxisome proliferator-activated receptor γ (PPAR-γ). Functional assays suggested that acetate production by *B. pseudolongum* contributes to competitive inhibition of EcNC101 growth.

**Conclusions:**

This study highlights the potential of microbiota-modifying interventions, particularly probiotics such as *B. pseudolongum*, to control pathobionts, reduce inflammation, and improve intestinal health in ulcerative colitis.

## Graphical Abstract

**Figure:**
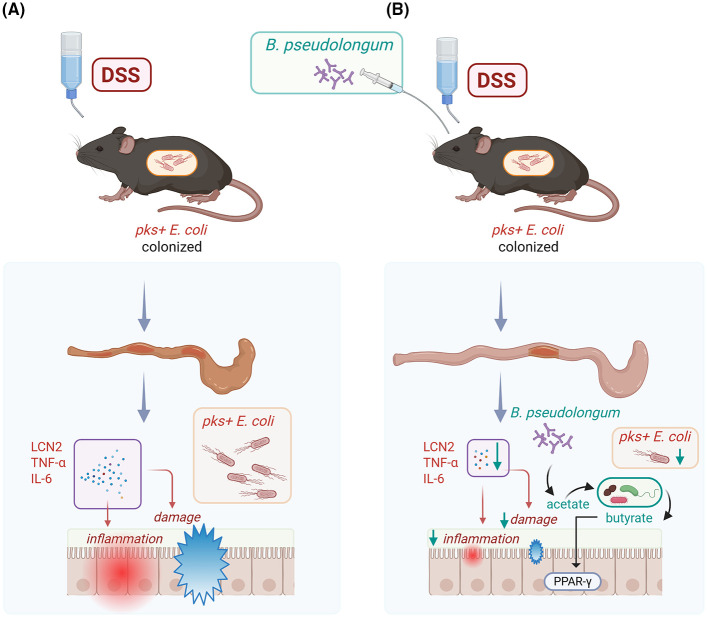
B. pseudolongum administration attenuates DSS-induced colitis in mice colonized with pks+ E. coli. (A) DSS-treated mice colonized with pks+ E. coli develop severe colitis characterized by decreased colon length, enhanced inflammation (LCN-2, TNF-α, IL-6), causing significant epithelial damage. (B) B. pseudolongum administration limits pks+ E. coli expansion during DSS administration through enhanced acetate production resulting in reduced inflammation and diminished epithelial damage. Acetate is additionally used by butyrate-producing bacteria thus enhancing butyrate production (cross-feeding) and resulting in the activation of the peroxisome proliferator-activated receptor γ (PPAR-γ).

## 1 Introduction

Ulcerative colitis is a chronic inflammatory bowel disease (IBD) characterized by inflammation of the mucosa of the colon and rectum (Ungaro et al., 2017). It affects millions of people worldwide and is associated with increased morbidity and risk of colorectal cancer (CRC) (Axelrad et al., 2016). Growing evidence implicates the gut microbiota as a key contributor to disease onset and progression (Füchtbauer et al., 2025). Imbalance of gut microbiota composition and function, or dysbiosis, in ulcerative colitis is characterized by reduced microbial diversity, loss of beneficial commensals, and expansion of potentially harmful bacteria (Franzosa et al., 2019).

Among potentially harmful microbes are pathobionts, defined as resident members of the normal microbiota that remain harmless under homeostatic conditions but acquire pathogenic properties in the context of barrier disruption or immune imbalance (Gilliland et al., 2024). Pathobionts exploit inflammatory or metabolic changes in the host to expand and may thus amplify disease processes (Zeng et al., 2017). Expansion of intestinal pathobionts has been associated with worsening inflammation and epithelial damage in IBD (Lawing and Bleich, 2025).

A particularly well-studied pathobiont in ulcerative colitis and CRC is *Escherichia coli* harboring the *pks* genomic island (*pks*+ *E. coli*). The *pks* island encodes colibactin, a peptide–polyketide genotoxin capable of inducing DNA double-strand breaks in eukaryotic cells (Nougayrède et al., 2006). Colibactin-associated mutational signatures have been detected in human colorectal tumors (Pleguezuelos-Manzano et al., 2020), indicating that chronic exposure to pks+ *E. coli* can contribute to carcinogenesis. Importantly, *pks*+ *E. coli* are enriched in the intestinal mucosa of ulcerative colitis patients compared to healthy individuals (López-Siles et al., 2022; Martin et al., 2004).

Microbiota-modifying interventions, including dietary interventions and probiotics, represent a promising strategy for restricting the expansion of genotoxic bacteria (Buffie and Pamer, 2013). In this study, we investigated whether microbiota-targeted interventions can limit *pks*+ *E. coli* expansion and attenuate disease severity in dextran sulfate sodium (DSS)-induced colitis in mice.

## 2 Materials and methods

### 2.1 Animal experiments

Animal studies were performed following the Canadian Council of Animal Care guidelines, after approval by the Institutional Animal Care Committee of the Center de recherche du Center hospitalier de l'Université de Montréal (CRCHUM). C57BL/6 mice purchased from Charles River laboratory (Saint Constant, QC, Canada), were bred under specific pathogen-free conditions. All mice used were females between 8 and 10 weeks old at the beginning of the experiments and were maintained on a 12-h light/dark cycle with unlimited access to food (Inotiv Teklad Diets, TD2018, IN, USA) and water.

### 2.2 Bacterial preparation and culture

The *E. coli strain* NC101 (EcNC101) used in this study was a gift from Dr. Christian Jobin (Cancer Microbiota and Host Response, UF Health Cancer Center, University of Florida). The bacteria was grown from glycerol stocks in lysogeny broth (LB) overnight at 37 °C with shaking at 150 revolutions per minute (rpm). The cultures were centrifuged at 9,500 x *g* for 1 min, and appropriate bacterial suspension was prepared in 0.9% sterile saline (Baxter, Mississauga, ON, Canada) for oral gavage in mice. *Bifidobacterium pseudolongum* subsp. *pseudolongum* DSM 20099 (Mitsuoka, 1969; Nouioui et al., 2018) was obtained from the German Collection of Microorganisms and Cell Cultures GmbH, and grown on tryptone soy agar plates with 5% sheep blood (Oxoid, Thermo Fischer Scientific inc., ON, Canada) at 37 °C in anaerobic conditions for 48 h. Bacterial colonies were scraped and suspended in sterile 0.9% saline (Baxter) for same-day administration by gavage. *B. pseudolongum* DSM 20099 culture identification was monitored by real-time quantitative polymerase chain reaction (qPCR) using primers (forward-5′ CAA GGC CAT CAA CTG GTT CA3′ and reverse-5′ ACG TCG TGC TGC TCG AAT GT3′), and by matrix-assisted laser desorption ionization time-of-flight (MALDI-TOF) mass spectrometry. Bacterial presence in mouse fecal samples was confirmed by qPCR.

### 2.3 EcNC101 colonization, DSS administration, and putrescine supplementation

Mice were first treated with a cocktail of antibiotics (ampicillin-0.25 mg/mL, streptomycin-0.75 mg/mL, and colistin-1 mg/mL) in drinking water for 3 days. One day after ending antibiotic treatment, mice received an oral gavage of 200 μL of EcNC101 bacterial suspension (10^8^ CFUs) in sterile sodium chloride solution (0.9% saline). Acute colitis was induced using 2.0–2.5% DSS in sterile water for 12 days (DB001; TdB Labs, Upsala, Sweden), while control groups received sterile water only. Where indicated, mice were treated with 1% dihydrochloride putrescine (P5780; Sigma-Aldrich), a concentration previously shown to suppress *pks*+ *E.coli* expansion *in-vivo* without observable toxicity in C57BL/6 mice or 1 % hydrochloride in drinking water during DSS treatment (Oliero et al., 2024). Both weight and a disease activity index (DAI) were evaluated daily, with DAI being scored based on the following scale: stool consistency (normal = 0; loose = 2; diarrhea = 4); rectal bleeding (none = 0; occult blood = 2; rectal bleeding = 4) (Chassaing et al., 2014; Kim et al., 2012; Ajayi et al., 2025). The presence of prolonged watery feces in the colon and the absence of fecal pellet formation were employed to describe diarrhea, whereas the difference between initial and testing weights was used to determine weight loss. Bleeding was assessed by hemoccult sensa (Beckman Coulter Inc., Brea, CA, USA). Mice were euthanized on day 12 by cervical dislocation and intraperitoneal injection of sodium pentobarbital. The colon and feces were collected, snap frozen in liquid nitrogen before final transfer to −80 °C for storage prior other assays.

### 2.4 Isolation of *E. coli* in fecal samples

Fecal homogenates were prepared from about 20 mg of fecal sample (one pellet) dissolved in 1mL sodium chloride (0.9%). Appropriate serial dilution of fecal homogenate was plated on MacConkey agar (Thermo Scientific Oxoid) and incubated overnight at 37 °C, after which the colonies were counted.

### 2.5 Protein quantification and ELISA

Homogenous suspension of fecal samples was first prepared by digesting about 20–30 mg of feces in 200 μL of radioimmunoprecipitation assay (RIPA) buffer that contained NaCl (150 mM), NP-40 (1%), deoxycholic acid (0.50%), SDS (0.10%), Tris pH 8.0 (50 mM), and protease inhibitors (cOmplete^TM^, Mini, EDTA free Protease Inhibitor Cocktail Roche, Mannheim, Germany). After that, the suspension was centrifuged (12,000 × g) for 5 min at 4 °C. Prior to analysis, an aliquot of the supernatant was made in a separate tube and kept at −20 °C. The Pierce^TM^ BCA Protein Assay Kit (Thermo Fisher, Rockford, IL, USA) was used to measure protein concentrations. Phosphate buffered sodium (PBS) containing 0.1% Tween 20 (reagent diluent) was used to prepare sufficient dilutions for ELISA. Afterwards, the ELISA MaxTM standard set mice IL-6 and TNF-α (BioLegend, San Diego, CA, USA) and the mouse lipocalin-2/NGAL ELISA kit (R&D Systems, Minneapolis, MN, USA) were utilized. The absorbance of the plates was measured using a multimode microplate reader (Tecan Spark, Morrisville, USA).

### 2.6 Histology

Colon histology was carried out as previously reported (Ajayi et al., 2025). Briefly, each mouse's proximal and distal colon sections were first preserved in 10% formalin (ChapTec, Montreal, QC, Canada). After that, the samples were embedded in paraffin and sectioned at a thickness of 4 μm. The sections were stained with hematoxylin (RICCA, VWR International, Mississauga, ON, Canada) and eosin (H&E; Leica Biosystems Richmond Inc. Richmond, IL, USA) to determine the level of colitis. A blinded histological analysis was performed evaluating the degree of epithelial cell loss (0–3), crypt damage (0–3), and inflammatory cell infiltration (0–3).

### 2.7 DNA Extraction, 16S rRNA Gene sequencing, and microbiota analysis

Fecal bacterial DNA was extracted using the DNeasy PowerSoil Pro Kit (QIAGEN, Hilden, Germany), following the manufacturer's instructions. Amplification and sequencing of the V5–V6 region of the 16S rRNA gene were performed at Genome Québec sequencing facility (Forward primer: P609D GGMTTAGATACCCBDGTA Reverse primer: P699R GGGTYKCGCTCGTTR). Sequencing was conducted on an Illumina NextSeq platform, generating 300 bp demultiplexed paired-end reads. Primer sequences were removed from raw sequencing reads using Cutadapt v4.8 (Martin, 2011). Amplicon sequence variant (ASV) inference was performed using the DADA2 pipeline v1.18 (Callahan et al., 2016) in R v4.3.0 generating a total of 3094 ASVs. Taxonomic assignment of ASVs was carried out using the SILVA v138.1 reference database (McLaren and Callahan, 2021). Alpha diversity was calculated using the Chao1, Inverse Simpson, and Shannon indexes, implemented in the Phyloseq package v1.52 (McMurdie and Holmes, 2012). Beta diversity was assessed using Principal Coordinate Analysis (PCoA) based on weighted Unifrac dissimilarity matrices calculated based on relative abundance-transformed data and an associated phylogenetic tree. To reduce noise and improve detection of biologically relevant signals, ASVs were filtered prior to differential abundance analyses. ASVs were excluded if they had fewer than 10 total counts across all samples or were present in fewer than 5% of samples. Differential abundance testing was performed on these ASVs using DESeq2 v1.48.1 (Love et al., 2014). Taxonomic relative abundances were computed from raw counts and visualized at the phylum and family levels using the R package StackbarExtended (Cuisiniere and Santos, 2024).

### 2.8 Peroxisome proliferator activated receptor gamma (PPARγ)

Colonic tissue was homogenized in lysis buffer, and its protein concentration was quantified using a BCA protein assay (Pierce™ BCA Protein Assay Kit). The activation of the PPAR-γ transcription factor was carried out on colonic homogenates using the TransAM DNA-binding ELISA kit following the manufacturer's instructions (Active Motif, Carlsbad, California, USA).

### 2.9 Short chain fatty acids and Bile acids

Quantification of SCFAs and BAs was performed using electrospray ionization mass spectrometry at the metabolomics core facility of the CRCHUM, as previously reported (Hajjar et al., 2021). Utilizing a polypropylene pestle, samples weighing about 20 mg were manually homogenized in 50% aqueous acetonitrile (30 μL per mg sample) and properly mixed for 5 min at 4 °C. Following centrifugation at 20,000 × g for 15 min at 4 °C, 10 μL of a 50% aqueous acetonitrile solution containing deuterated internal standards was added to glass tubes along with 30 μL of supernatants, blanks, and standards. 3-nitrophenylhydrazine was used to derivatize carboxyl groups. Derivatized organic acids were identified by ESI–MS/MS in negative-ion mode (QTRAP 6500, SCIEX) after being separated by reversed-phase chromatography (Nexera X2, Shimadzu) with the use of a C18 column (Poroshell 120 EC-C18, 2.1 × 75 mm, 2.7 μm, Agilent).

### 2.10 EcNC101 growth with *B. pseudolongum* supernatant

*B. pseudolongum* (Bp) was cultured anaerobically overnight in modified peptone yeast glucose (PYG) medium at 37 °C. The following day, bacterial growth was monitored by measuring optical density (OD600), and cultures were harvested at an OD600 of approximately 0.5. Cells were pelleted by centrifugation at 300 × *g* for 10 min at room temperature, and the resulting supernatant was collected and filtered through a 0.22 μm pore-size membrane to remove residual bacterial cells. The filtered preparation was designated as the *B. pseudolongum* supernatant (Bp SN). Control supernatant (Ctrl SN) was prepared in parallel using sterile PYG medium that underwent the same incubation, centrifugation, and filtration steps, but without bacterial inoculation. Both supernatants were stored at −80 °C until use. For growth inhibition assays, EcNC101 was cultured overnight in PYG medium under identical anaerobic conditions. Bp SN and Control (Ctrl) SN were diluted in fresh PYG to obtain final concentrations of 25%, 50%, and 100%. Each treatment condition (200 μL per well) was inoculated with 1 × 107 CFU/mL of EcNC101 and dispensed in triplicate into a sterile, clear 96-well microplate. Plates were incubated anaerobically at 37 °C, and bacterial growth was monitored by measuring OD600 every hour using a Stratus Kinetic Microplate Reader (Cerillo, Charlottesville, VA, USA).

### 2.11 Statistics

Graphpad Prism (Version 10.4.1), Graphpad software, San Diego, CA, USA) was used to analyze all data. Shapiro-Wilk was used for data normality check, while F test (two variances) and Barlett's test (multiple variances) were used to check for homogeneity of variance. When the data did not pass the Shapiro–Wilk normality test, log(Y) transformation was applied to the data. Statistical significance was determined at *P-*values less than 0.05.

## 3 Results

### 3.1 Colonization with EcNC101 exacerbates chemically-induced acute colitis

To model host-pathobiont interactions relevant to ulcerative colitis, we used EcNC101, a murine adherent-invasive *E. coli* (AIEC) strain that causes aggressive colitis in interleukin-10-deficient mice (Kim et al., 2005). EcNC101 shares several features with AIEC strains associated with human IBD and CRC (Martin et al., 2004; Shen et al., 2010).

**Figure 1:**
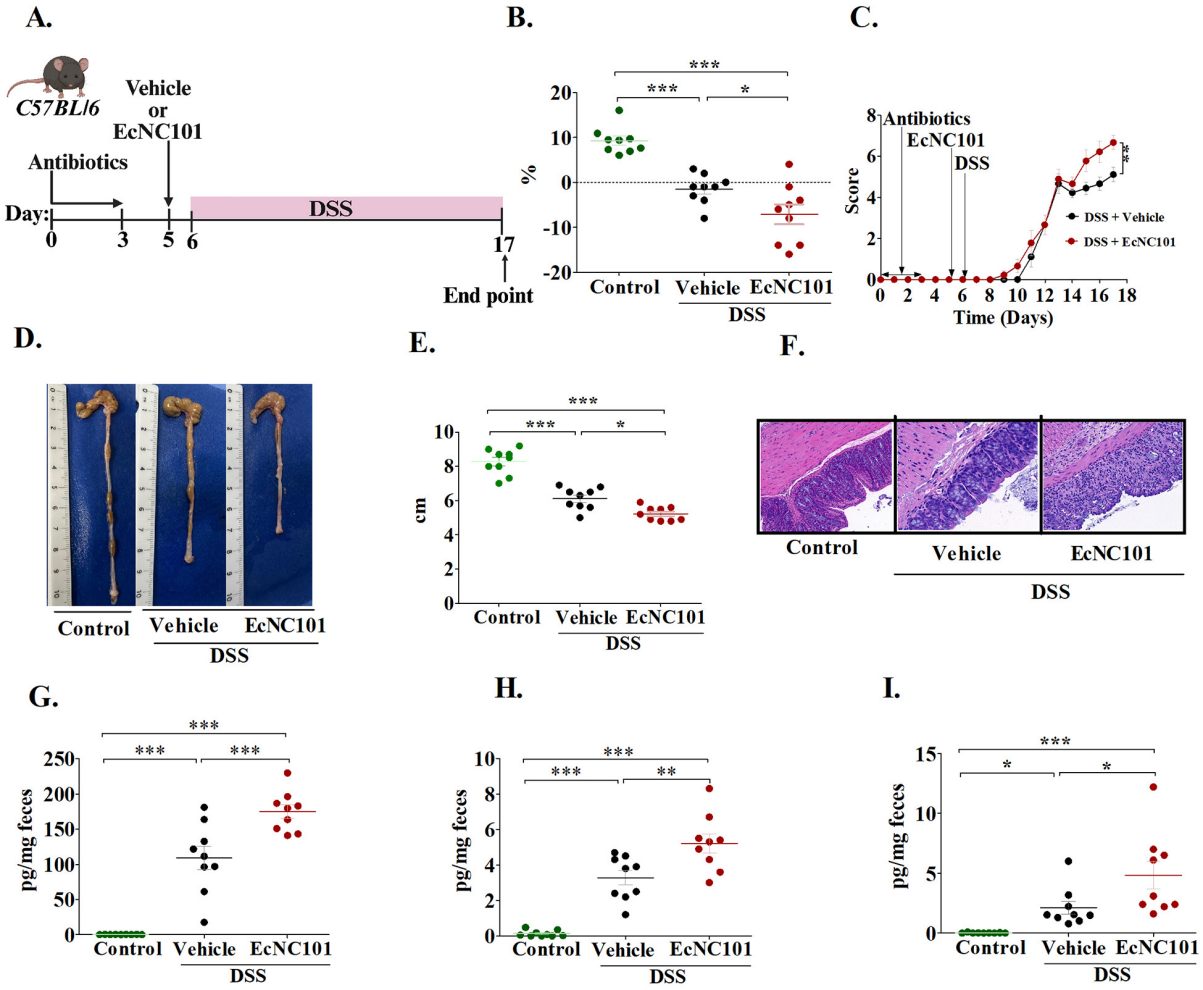
EcNC101 colonization exacerbates DSS-induced colitis. **(A)** Study design. **(B)** Body weight variation. **(C)** Disease activity index. **(D)** Representative images of mouse colons. **(E)** Colon length. **(F)** Representative H&E staining images of the colon tissues in mice (200x). **(G–I)** Fecal levels of LCN-2 **(G)**; IL-6 **(H)**; and TNF-α **(I)**. Each dot represents one mouse, and means are represented by horizontal bars ± SEM; *n* = 9 mice per group. ANOVA ^*^P < 0.05, **P < 0.01, ***P < 0.001. DSS, dextran sodium sulfate; EcNC101, *Escherichia coli* NC101. Disease activity index was scored as stool consistency (normal = 0; loose = 2; diarrhea = 4) plus rectal bleeding (none = 0; occult blood = 2; rectal bleeding = 4).

C57BL/6 mice were treated with antibiotics to reduce beneficial microbes and hence overcome colonization resistance, after which they were colonized with EcNC101 and treated with DSS to induce acute colitis (Figure 1A). As shown in Figure 1, EcNC101-colonized mice developed more severe colitis as judged by body weight loss, DAI scores, and shortening of the colon (Figures 1B–E and Supplementary Table S1). DSS treatment caused visible changes in pathological parameters including crypt distortion, epithelial damage, ulceration, and inflammatory cell infiltration. The overall histological damage was more severe in EcNC101-colonized mice compared to the non-colonized mice, and no pathological distortion was observed in the control mice (Figure 1F and Supplementary Figure S1). Accordingly, fecal levels of LCN-2, IL-6 and TNF-α were also significantly higher in mice colonized with EcNC101 (Figures 1G–I).

These results show that colonization with EcNC101 aggravates DSS-induced colitis in mice, providing a convenient model to investigate how microbiota-modifying interventions influence pathobiont expansion and acute inflammatory responses.

### 3.2 Treatment with putrescine improves DSS-induced colitis in mice colonized with EcNC101

**Figure 2:**
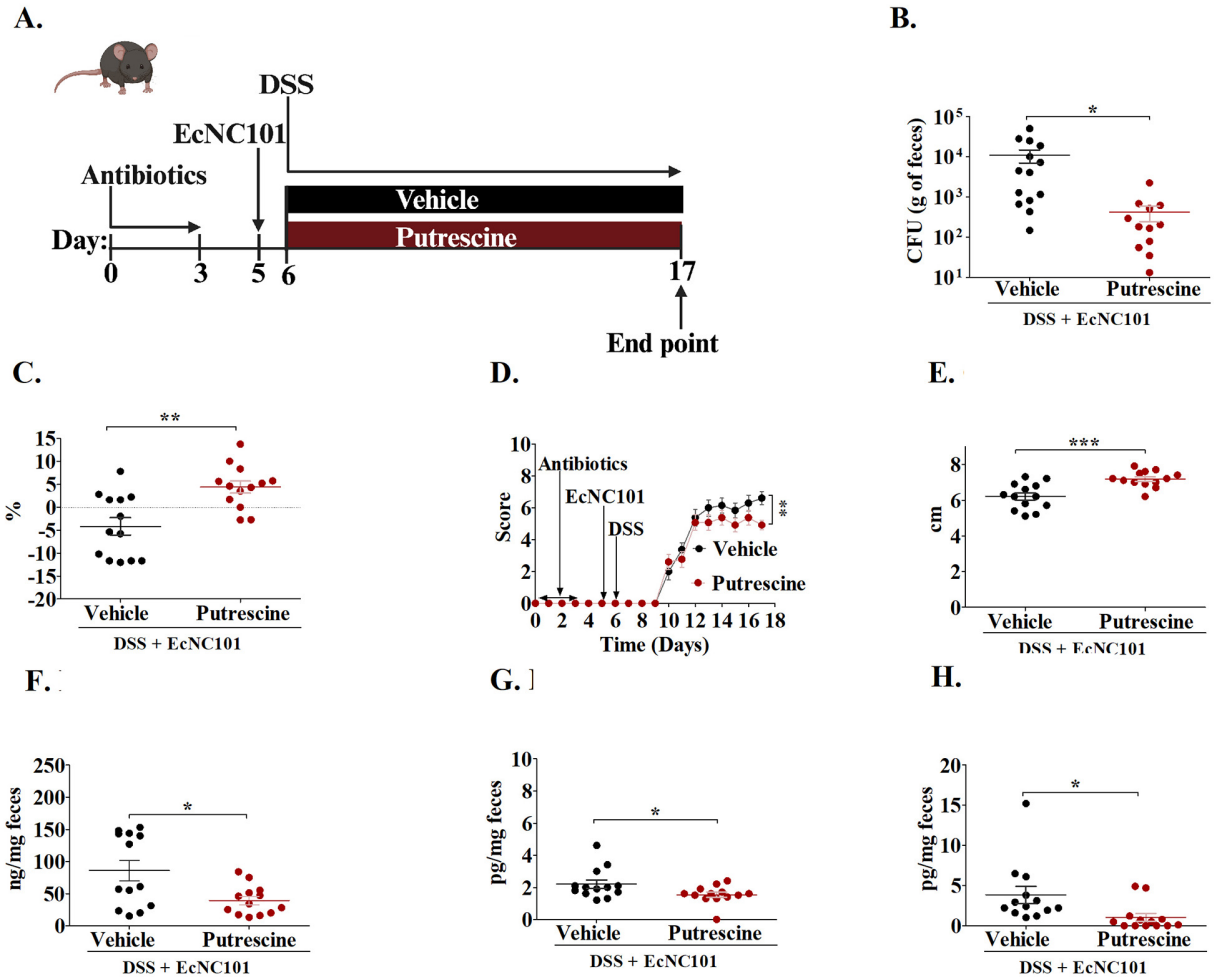
Reduction of EcNC101 expansion by dietary putrescine alleviates colitis severity. **(A)** Study design. **(B)** EcNC101 colonization. **(C)** Body weight variation. **(D)** Disease activity index. **(E)** Colon length. **(F–H)** Fecal levels of LCN-2 **(F)**; IL-6 **(G)**; and TNF-α **(H)**. Each dot represents one mouse, and means are represented by horizontal bars ± SEM; *n* = 13 mice per group. Student *t*-test, *P < 0.05, **P < 0.01, ***P < 0.001. DSS, dextran sodium sulfate; EcNC101, *Escherichia coli* NC101. Disease activity index was scored as stool consistency (normal = 0; loose = 2; diarrhea = 4) plus rectal bleeding (none = 0; occult blood = 2; rectal bleeding = 4).

Polyamines such as putrescine support gut epithelial integrity and barrier function (Nakamura and Matsumoto, 2025) and can modulate the virulence and colonization dynamics of bacterial pathogens (Shah and Swiatlo, 2008). Previously, we identified putrescine as a potential postbiotic capable of suppressing EcNC101 growth *in vitro* and *in vivo* in the context of colorectal cancer (Oliero et al., 2024). To further evaluate the potential protective effect of putrescine during acute colitis in mice colonized with EcNC101, putrescine was added to the drinking water during DSS treatment (Figure 2A). Putrescine supplementation significantly suppressed EcNC101 growth compared to vehicle-treated mice (Figures 2B) and attenuated colitis as evidenced by higher body weights (Figure 2C and Supplementary Table S2), reduced DAI scores (Figure 2D), and longer colons (Figure 2E). Furthermore, putrescine caused a significant reduction in fecal LCN-2, IL-6, and TNF-α (Figures 2F–H).

Together, these data indicate that putrescine supplementation effectively attenuates colitis in EcNC101-colonized mice.

### 3.3 Putrescine supplementation alters gut microbial composition and function in mice colonized with EcNC101

**Figure 3:**
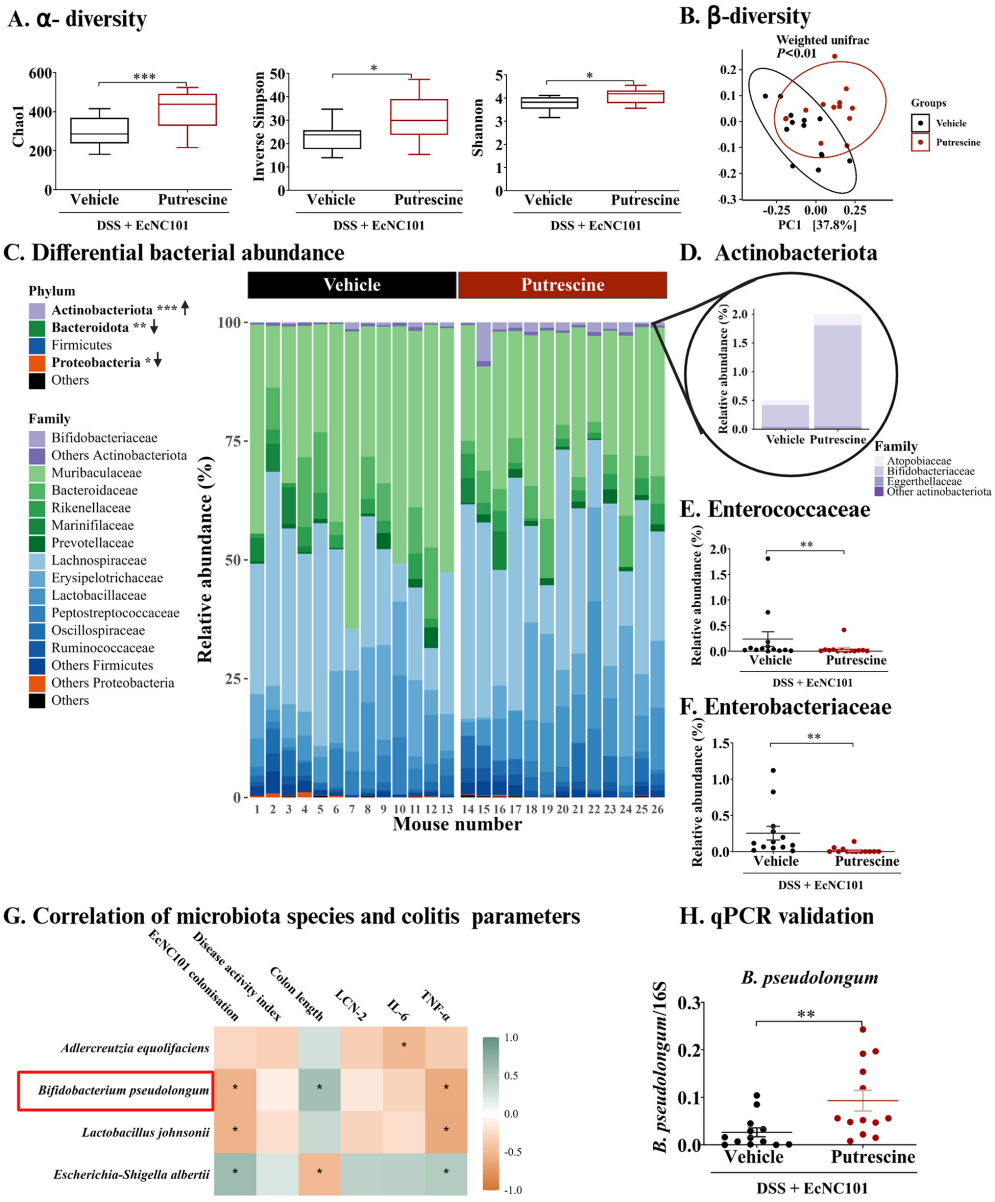
Putrescine supplementation modulates gut microbiota composition in mice. **(A)** Alpha diversity metrics: Chao1, Inverse Simpson, and Shannon diversity indices shown as boxplots (Student's *t*-test). **(B)** Principal coordinate analysis (PCoA) of microbial communities based on weighted UniFrac distances (ADONIS). **(C)** Differential bacterial abundance at the phylum and family levels (DESeq2 Wald test, FDR corrected; each stacked bar represents a single mouse; ↑↓ indicate increase or decrease in abundance in the putrescine group relative to the vehicle group). **(D)** Actinobacteriota relative abundance. **(E,F)** Family: Enterococcaceae **(E)**; and Enterobacteriaceae **(F)** (DESeq2 Wald test, FDR corrected). **(G)** Correlation matrix of best putrescine predictive microbiota features and colitis parameters (Spearman's rank correlation test, FDR-adjusted). **(H)** *B. pseudolongum* quantified by real-time PCR (Student's *t-*test). Each dot represents one mouse, and means are represented by horizontal bars ± SEM; *n* = 13 mice per group. DSS = dextran sodium sulfate. *P < 0.05, **P < 0.01, ***P < 0.001.

Because putrescine supplementation attenuated the exacerbated colitis caused by EcNC101 colonization, we questioned whether the bacterial community as a whole was altered by putrescine in EcNC101-colonized mice. To investigate this, we performed 16S rRNA sequencing to characterize the gut microbiota in these mice. α-diversity analysis revealed that putrescine supplementation significantly increased microbial diversity (Figure 3A). Furthermore, unconstrained principal coordinate analysis (PCoA) showed significantly different clustering of the fecal microbiota between mice treated with putrescine and those that received the vehicle (Figure 3B; *P* < 0.01). Differential abundance analysis was then performed between the vehicle and putrescine groups. At the phylum level, the relative abundance of Actinobacteriota was increased, while the relative abundance of Proteobacteria and Bacteroidota decreased (Figure 3C). The increase in Actinobacteriota relative abundance was driven mostly by an expansion of Bifidobacteria (Figure 3D). Two families were significantly reduced (Figures 3E, F), namely *Enterococcaceae* (phylum Firmicutes) and *Enterobacteriaceae* (phylum Proteobacteria). Several amplicon sequence variants (ASVs) were found to be significantly different (Supplementary Figure S2), among which four were annotated at the species level. We performed correlations with these four ASVs and identified an ASV annotated as *Bifidobacterium pseudolongum* (phylum Actinobacteria) that was negatively correlated with EcNC101 colonization and colonic TNF-?? while positively associated with colon length (Figure 3G), and was thus designated as potentially beneficial. Finally, using quantitative polymerase chain reaction (qPCR), we confirmed that mice treated with putrescine had a higher abundance of *B. pseudolongum* compared to vehicle-treated mice (Figure 3H).

**Figure 4:**
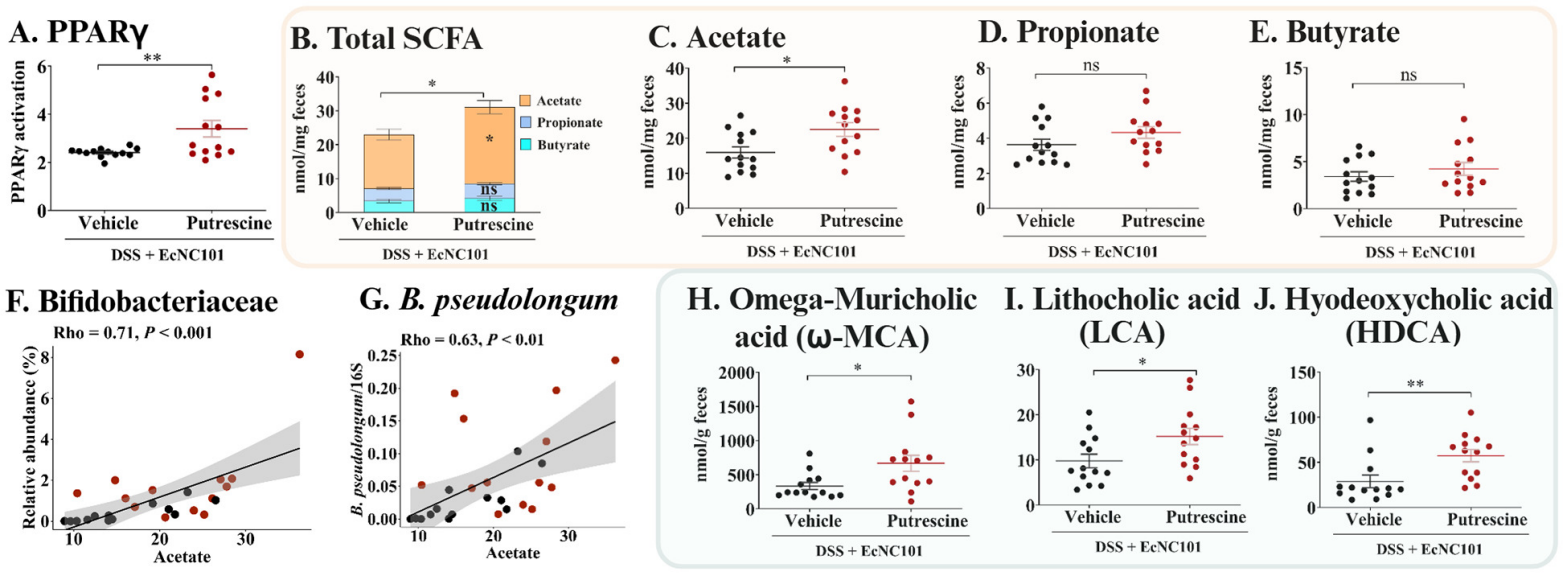
Putrescine supplementation impacts gut microbiota function in mice. **(A)** Peroxisome proliferator-activated receptor gamma (PPARγ) activation. **(B-E)** Short-chain fatty acids: total SCFA **(B)**; acetate **(C)**; propionate **(D)**; and butyrate **(E)**. **(F)** Correlation between fecal acetate levels and relative abundance of Bifidobacteriaceae **(G)** Correlation between fecal acetate levels and the abundance of *B. pseudolongum* assessed by qPCR (Spearman's rank correlation test). **(H-J)** Bile acids: omega-muricholic acid (ω-MCA) **(H)**; lithocholic acid (LCA) **(I)**; and hyodeoxycholic acid **(J)**. Each dot represents one mouse, and means are represented by horizontal bars ± SEM; *n* = 13 mice per group. Student's *t*-test, ^*^P < 0.05, **P < 0.01. DSS = dextran sodium sulfate.

Peroxisome proliferator activated receptor γ (PPARγ), a member of the nuclear receptor superfamily of transcription factors, is expressed in intestinal epithelial cells, and its microbiota-mediated activation during colitis has been shown to be protective (Adachi et al., 2006). We found that PPARγ activation was significantly enhanced in mice receiving putrescine (Figure 4A), suggesting that putrescine supplementation may additionally impact gut microbiota metabolites. We therefore used targeted metabolomics and found that indeed putrescine supplementation significantly enhanced the production of total SCFA, with this increase mostly due to acetate (Figures 4B, C), but not propionate or butyrate (Figures 4D, E and Supplementary Figure S3). Furthermore, acetate levels positively correlated with the relative abundance of Bifidobacteriaceae (Rho = 63, *P* < 0.0001; Figure 4F) and *B. pseudolongum* assessed by qPCR (Rho = 63, *P* < 0.001; Figure 4G). In addition, the bile acids omega-muricholic acid (ω-MCA), lithocholic acid (LCA), and hyodeoxycholic acid (HDCA) were significantly increased in putrescine-treated mice (Figures 4H–J and Supplementary Figure S4).

When considered together, these findings suggest that putrescine supplementation modulates the composition and function of the gut microbiota and that *B. pseudolongum* may play a central role in mitigating colitis in mice infected with EcNC101.

### 3.4 *Bifidobacterium pseudolongum* DSM 20099 inhibits EcNC101 growth and attenuates acute colitis in mice

**Figure 5:**
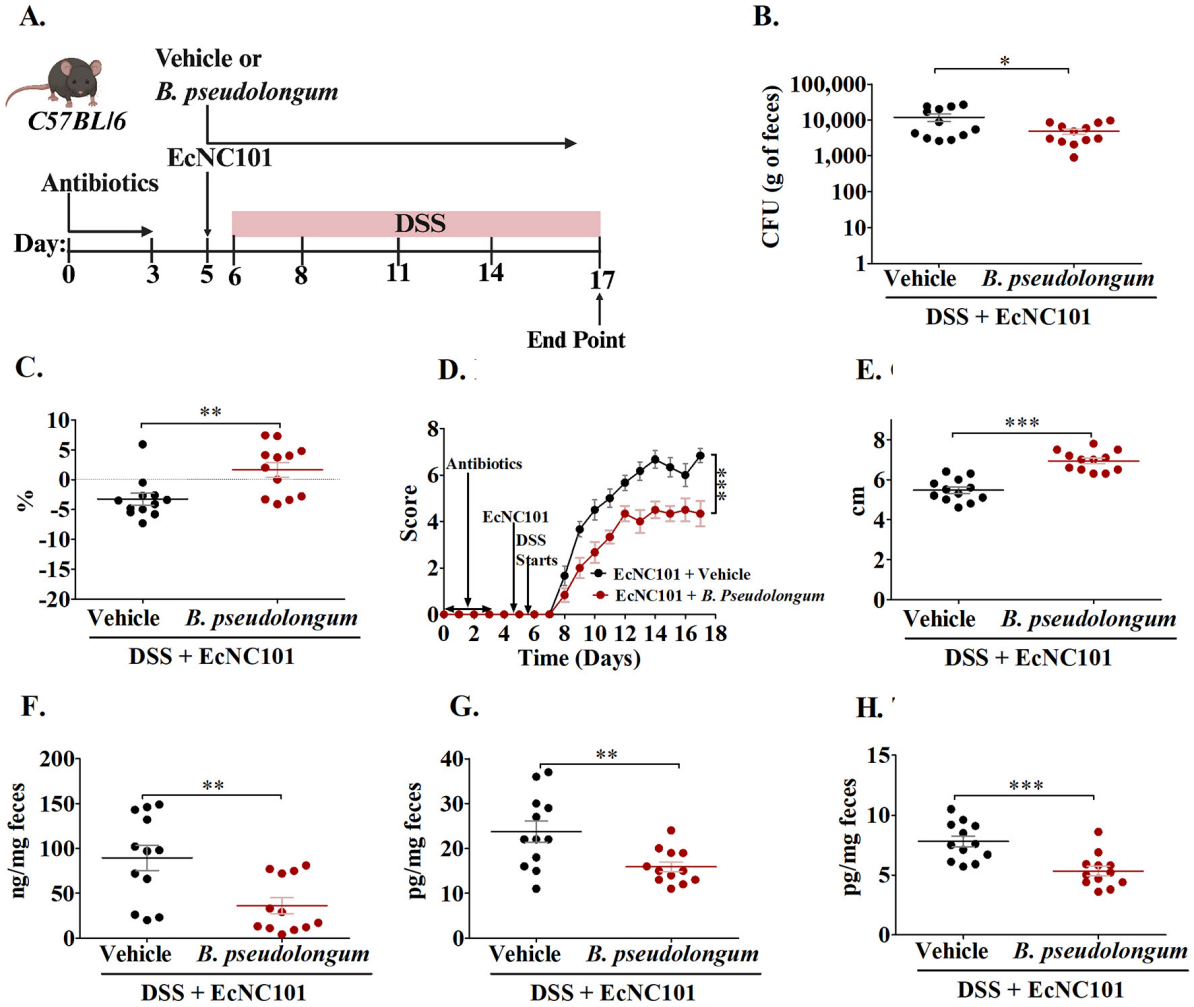
*B. pseudolongum* DSM 20099 inhibits EcNC101 and limits DSS-induced colitis. **(A)** Study design. **(B)** EcNC101 colonization. **(C)** Body weight variation. **(D)** Disease activity index. **(E)** Colon length. **(F-H)** Fecal levels of LCN-2 **(F)**; IL-6 **(G)**; and TNF-α **(H)**. Each dot represents one mouse, and means are represented by horizontal bars ± SEM; *n* = 12 mice per group. Student's *t-test*, ^*^P < 0.05, **P < 0.01, ***P < 0.001. DSS = dextran sodium sulfate, EcNC101 = *Escherichia coli* NC101. Disease activity index was scored as stool consistency (normal = 0; loose = 2; diarrhea = 4) plus rectal bleeding (none = 0; occult blood = 2; rectal bleeding = 4).

To further evaluate the potential therapeutic effect of *B. pseudolongum*, EcNC101-colonized mice were administered *B. pseudolongum* DSM 20099 daily during DSS treatment by oral gavage (Figure 5A). *B. pseudolongum* DSM 20099 shared 98% sequence identity of the variable V5–V6 region of the 16S rRNA gene with the identified *B. pseudolongum* ASV (Supplementary Figure S5). *B. pseudolongum* DSM 20099 administration significantly inhibited the growth of EcNC101 and significantly attenuated DSS-induced colitis, as evidenced by decreased weight loss, marked reduction in DAI scores, and attenuated colonic shortening (Figures 5B–D and Supplementary Table S3). In addition, a significant decrease in the levels of LCN-2, IL-6, and TNF-α in fecal samples of the *B. pseudolongum*-treated group was found (Figures 5F–H).

**Figure 6:**
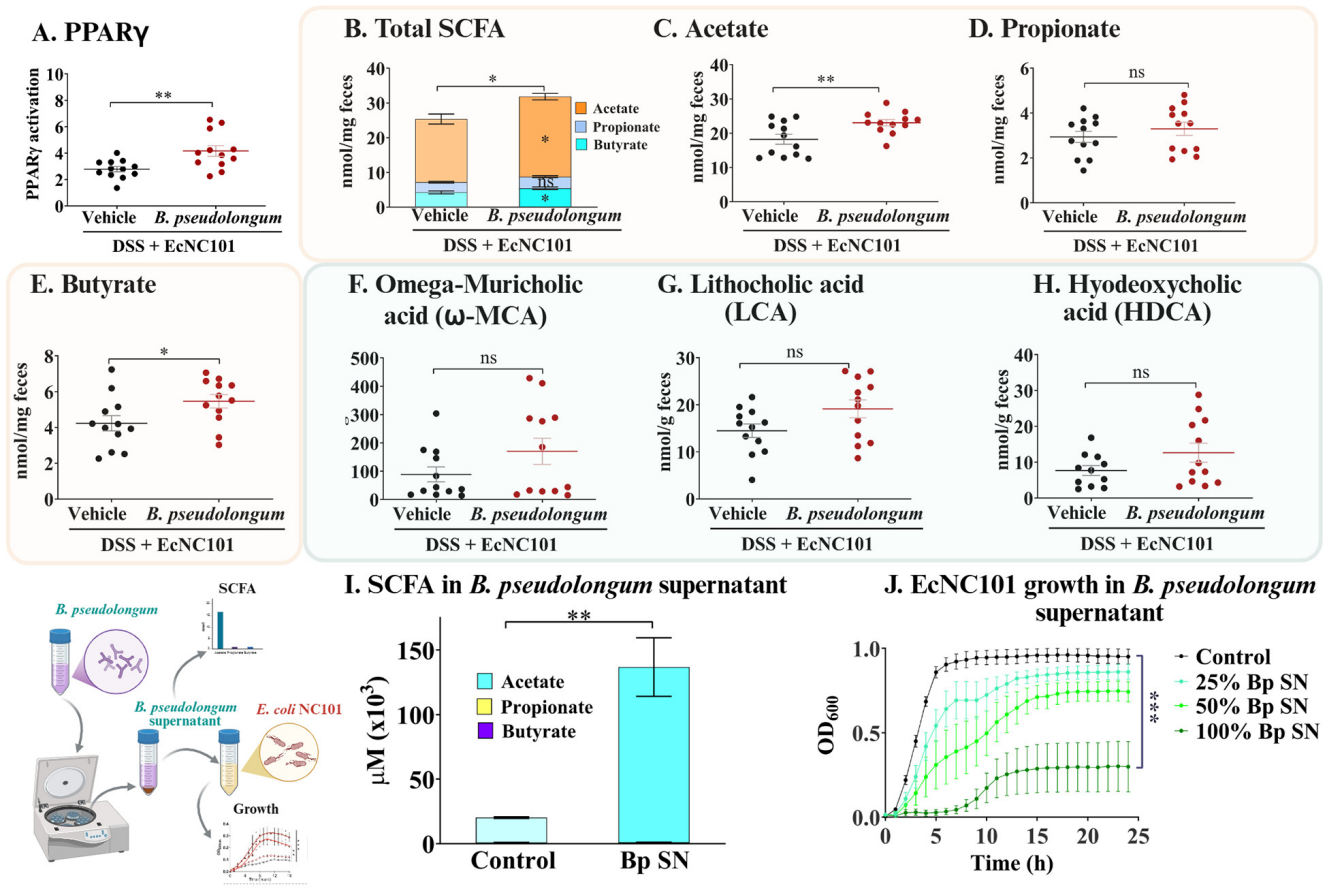
*Bifidobacterium pseudolongum* DSM 20099 promotes SCFA production to limit EcNC101 growth. **(A)** Peroxisome proliferator-activated receptor gamma (PPARγ) activation. **(B–E)** Short-chain fatty acids: total SCFA **(B)** acetate **(C)**; propionate **(D)**; and butyrate **(E)**. **(F–H)** Bile acids: omega-muricholic acid (ω-MCA) **(F)**; lithocholic acid (LCA) **(G)**; and hyodeoxycholic acid **(H)**. **(I)** SCFA in *B. pseudolongum* supernatant. **(J)** EcNC101 growth in *B. pseudolongum* supernatant. Each dot represents one mouse, and means are represented by horizontal bars ± SEM; *n* = 12 mice per group. Student's *t*-test, ^*^P < 0.05, **P < 0.01, ***P < 0.001. DSS = dextran sodium sulfate, Bp SN = *B. pseudolongum* supernatant.

To investigate the impact of oral administration of *B. pseudolongum* DSM 20099 on targeted bacterial metabolites, SCFA and bile acids were quantified in fecal samples obtained from mice receiving *B. pseudolongum* or saline as a control. *B. pseudolongum* DSM 20099 administration activated PPARγ and significantly increased the production of acetate and butyrate (Figures 6 and Supplementary Figures S6, S7).

To further confirm that *B. pseudolongum* DSM 20099 is an acetate-producer, we assessed SCFA in supernatants from bacteria grown in PYG medium. Acetate, but not butyrate nor propionate, were abundant in *B. pseudolongum* supernatants (Figure 6I). Importantly, *B. pseudolongum* supernatants reduced EcNC101 growth relative to control supernatant across all dilutions tested (Figure 6J), providing preliminary *in vitro* evidence consistent with a role for *B. pseudolongum*-derived metabolites in limiting EcNC101.

Taken together, these results show that oral administration of *B. pseudolongum* DSM 20099 limits EcNC101 expansion *in vitro* and *in vivo* and effectively mitigates colitis severity in mice infected with this pathobiont, most likely via the production of acetate. Although acetate is a plausible contributor, other metabolites or mechanisms may also be involved, and their causal contributions remain to be experimentally demonstrated.

## 4 Discussion

Pathobionts are commensal microbes that can acquire pathogenic behavior when the balanced intestinal ecosystem is disrupted, such as during acute colitis. Upon expansion, these organisms can amplify inflammation, compromise epithelial integrity, and perpetuate disease progression (Chandra et al., 2021). Here we demonstrate that putrescine and *B. pseudolongum* DSM 20099 supplementation alleviates DSS-induced colitis in mice colonized with EcNC101, a genotoxic pathobiont associated with ulcerative colitis and colorectal cancer (Arthur et al., 2012).

Consistent with the ability of polyamines to modulate microbial metabolism and host signaling, putrescine administration altered both the composition and the function of the gut microbiota, as evidenced by PPAR-γ activation (Nakamura and Matsumoto, 2025). Microbiota-activated PPAR-γ signaling has been shown to prevent the expansion of potentially pathogenic *Escherichia* by reducing the bioavailability of respiratory electron acceptors to Enterobacteriaceae in the lumen of the colon (Byndloss et al., 2017). The increase in acetate levels strongly correlated with the enrichment of acetate-producing taxa such as Bifidobacteriaceae (belonging to the phylum Actinobacteria) (Devika and Raman, 2019). Concomitantly, the putrescine-mediated increase of secondary bile acids, namely ω-MCA, LCA, and HDCA, indicates enhanced microbial bile acid transformation of liver-derived primary bile acids. The shared anti-inflammatory properties of ω-MCA (Dempsey et al., 2019), LCA (Liu et al., 2025), and HDCA (Song et al., 2020), and the reported ability of HDCA to upregulate intestinal PPAR-γ expression (Lin et al., 2025) are relevant to ulcerative colitis and, in fact, are currently being investigated among other secondary bile acids for their potential use in targeted therapeutic approaches (Yang et al., 2021).

A key finding in this study was the identification of *B. pseudolongum* following 16S rRNA sequencing, which was significantly enriched in putrescine-treated mice and correlated with disease improvement. Mice orally administered *B. pseudolongum* DSM 20099 displayed reduced EcNC101 expansion and attenuated colitis severity, further supporting a potential role for this species in mediating, at least partially, the protective effects of putrescine supplementation. However, unlike putrescine, while *B. pseudolongum* oral administration selectively enhanced acetate and butyrate production, it did not increase secondary bile acid levels. Bifidobacteria have been reported to be acetate-producers (Fukuda et al., 2011), and we confirmed *B. pseudolongum* DSM 20099 to be an acetate-producer. However, since we did not detect butyrate in *B. pseudolongum* DSM 20099 supernatants, the increase in luminal butyrate likely occurs via cross-feeding between Bifidobacteria and butyrate-producing colonic bacteria (De Vuyst and Leroy, 2011).

Acetate, which is elevated by both putrescine supplementation and *B. pseudolongum* DSM 20099 administration, could mediate protection in colitis through both host-directed and microbe-directed mechanisms. For instance, acetate can act directly on host cells by binding mainly to G protein–coupled receptor (GPR) 43 (also known as free fatty acid receptor (FFAR) 2), which is abundantly expressed in intestinal epithelial cells, adipocytes, and immune cells (Yang et al., 2018). Activation of GPR43 by SCFA has been shown to play a pivotal role in the regulation of gut immunity and inflammatory responses, and is therefore highly relevant to IBD (Maslowski et al., 2009; Masui et al., 2013). Additionally, acetate can also act by inhibiting the growth and expansion of EcNC101 (Caballero-Flores et al., 2023), which is important considering that bacterial competition for both nutrients and space has been recognized as a fundamental mechanism in preventing opportunistic pathobiont expansion (Caballero-Flores et al., 2023). Consistent with this, *in vitro* exposure of EcNC101 to *B. pseudolongum* DSM 20099 supernatant markedly reduced its growth.

Compared to putrescine supplementation, *B. pseudolongum* offers several advantages as a therapeutic strategy. First, Bifidobacteria are among the most commonly used and safe human probiotics, and are certified by the Food and Drug Administration (FDA) as GRAS (Generally Recognized As Safe) and by the European Food Safety Authority (EFSA) as QPS (Qualified Presumption of Safety) (De Vuyst and Leroy, 2011). In fact, *Bifidobacterium* species are among the earliest colonizers of the human gastrointestinal tract and are widely recognized for their beneficial effects on the host (Derrien et al., 2022). Therefore, supplementation aims not to introduce a novel organism, but rather, to augment an existing beneficial member of the microbial community. In contrast to direct putrescine administration, which can have systemic effects and uncertain metabolic consequences, enhancing Bifidobacteria populations provides a microbiota-targeted strategy that sustains local acetate and butyrate production, supports epithelial integrity, and limits pathobiont expansion.

Translation of these findings to human ulcerative colitis raises several considerations. *Bifidobacterium*. species abundance in adult human gut microbiota decreases with age relative to infancy (Derrien et al., 2022; Odamaki et al., 2016; Gueimonde et al., 2007). Whether exogenous supplementation achieves durable colonization in the adult gut in the presence of an established resident gut microbiota has not been demonstrated and would require clinical investigation. Acetate concentrations sufficient for direct antimicrobial effects against *E. coli in-vivo* may exceed those produced by *B. pseudolongum* supplementation alone, suggesting that multi-strain or dietary co-interventions may be needed. Moreover, the composition of the resident microbiota, which varies substantially between individuals and disease states is likely to impact probiotic efficacy in ways that the present experimental design cannot model.

Some methodological limitations of the present study warrant consideration. Although EcNC101 carries the *pks* genomic island, the present study did not include measurements of colibactin production, *pks* gene expression, or colibactin-associated DNA damage markers such as gamma-H2AX foci. The observed suppression of EcNC101 expansion therefore does not establish direct inhibition of colibactin-mediated genotoxicity, which would require dedicated follow-up experiments. Furthermore, the DSS model recapitulates chemically induced epithelial barrier disruption and innate immune activation but does not fully replicate the chronic adaptive immune dysregulation, characteristic of human ulcerative colitis (Chassaing et al., 2014). Whether the protective effects of *B. pseudolongum* extend to immune-driven models, such as IL-10-deficient mice or T-cell transfer colitis, remains to be determined and represents an important direction for future works. As only female mice were used, potential sex-specific differences in microbiota composition, SCFA production, DSS susceptibility, and immune responses should be considered when interpreting these results. In addition, the microbiome analyses presented in this study focused on differential taxonomic abundance and its correlation with disease outcome parameters. Complementary approaches including linear discriminant analysis effect size (LEfSe), microbial network modeling, and metagenomics functional prediction could further characterize the ecological interactions underlying EcNC101 inhibition, and are warranted in future studies.

## 5 Conclusions

Our study demonstrates that dietary and probiotic interventions can effectively limit pathobiont expansion and mitigate inflammation in experimental colitis. Putrescine supplementation reshaped the gut microbiota, activating PPAR-γ-associated anti-inflammatory pathways and enhancing acetate and secondary bile acid production. In turn, enrichment with *B. pseudolongum* DSM 20099 selectively increased acetate and butyrate levels, which contributed to colitis mitigation and competitive reduction of EcNC101. These findings provide a conceptual and experimental framework for microbiota-based strategies to prevent or treat ulcerative colitis.

## Data Availability

Sequence data generated and analyzed during the current study are available in the NCBI SRA repository (BioProject PRJNA1373350) (https://www.ncbi.nlm.nih.gov/bioproject/PRJNA1373350).
