# Supplementary material for: Expansion resistance against genotoxic E. coli: protective effects of Bifidobacterium pseudolongum in DSS-induced colitis in mice
Source: Front Microbiol. 2026 Jul 28;17:1877186. doi: 10.3389/fmicb.2026.1877186 (PMC13457206; doi:10.3389/fmicb.2026.1877186)
Supplement: Supplementary file 1 [file Data_Sheet_1.docx]

Supplementary Material

# 1 Supplementary Data

## 1.1 Supplementary Figures

**Supplementary figure S1. Histopathological assessment of H&E of stained colon samples**. (A) Representative full colon H&E stained images; (B) Histological evaluation of colon samples. Scoring parameters: epithelial cell loss (0-3), crypt damage (0-3), and inflammatory cell infiltration (0-3). Each dot represents one mouse, and means are represented by horizontal bars ± SEM; n = 9 mice per group. ANOVA **P* < 0.05, ***P* < 0.01, ****P* < 0.001. DSS = dextran sodium sulfate, EcNC101= *Escherichia coli* NC101.

**Supplementary figure S2. Differential bacterial abundance in response to putrescine treatment.** Differential bacterial abundance at the ASV level (DESeq2 Wald test, FDR corrected). Dot represents Log2Fold change and bars represent Log2Fold change standard error. All *P* < 0.05.

**Supplementary figure S3. Fecal short chain fatty acid levels in response to putrescine supplementation.** Short chain fatty acids: isobutyrate (A); isovalerate (B); valerate (C); caproate (D); lactate (E); and succinate (F). Each dot represents one mouse, and means are represented by horizontal bars ± SEM; n=13 mice per group. Student’s *t*-test, **P <* 0.05. ns = non-significant, DSS = dextran sodium sulfate, EcNC101= *Escherichia coli* NC101.

**Supplementary figure S4. Fecal bile acid levels in response to putrescine supplementation.** (A-G) Bile acids: alpha-muricholic acid (α-MCA) (A); beta-muricholic acid (β-MAC) (B); Cholic acid (C); deoxycholic acid (DCA) (D); taurocholic acid (TCA) (E); taurochenodeoxycholic acid (TCDCA) (F); and taurodeoxycholic acid (TDCA) (G). Each dot represents one mouse, and means are represented by horizontal bars ± SEM; n = 13 mice per group. Student’s *t-*test, ns = non-significant, DSS = dextran sodium sulfate, EcNC101= *Escherichia coli* NC101.

# Supplementary figure S5. The variable V5-V6 region of a 16S rRNA gene of *Bifidobacterium pseudolongum* subsp. *pseudolongum* DSM 20099 is similar to *B. pseudolongum* ASV. Sequence alignment of *B. pseudolongum* ASV with *B. pseudolongum* DSM 20099 16S rRNA gene copy variant using NCBI BLASTn.

**Supplementary figure S6. The effect of *B. pseudolongum* on fecal short chain fatty acid levels.** (A-F) Short chain fatty acids: Isobutyrate (A); Isovalerate (B); Valerate (C); Caproate (D); Lactate (E); and Succinate (F). Each dot represents one mouse, and means are represented by horizontal bars ± SEM; n = 12 mice per group. Student’s *t*-test, ns = non-significant, DSS = dextran sodium sulfate, EcNC101= *Escherichia coli* NC101.

 (A)**Supplementary figure S7. Fecal bile acid levels in mice administered oral *B. pseudolongum*.** (A-H) Bile acids: alpha-muricholic acid (α-MCA) (A); beta-muricholic acid (β-MAC) (B); cholic acid (C); ursodeoxycholic acid (UDCA) (D); deoxycholic acid (DCA) (E); taurocholic acid (TCA) (F); taurochenodeoxycholic acid (TCDCA) (G), and taurodeoxycholic acid (TDCA) (H). Each dot represents one mouse, and means are represented by horizontal bars ± SEM; n = 12 mice per group. Student’s *t*-test, ns = non-significant, DSS = dextran sodium sulfate, EcNC101= *Escherichia coli* NC101.

**Supplementary table S1. Raw body weight data of DSS-treated EcNC101-colonized mice**

| **Groups** | **Initial body weight (g)** | **Final body weight (g)** | **Body weight variation (%)** |
| --- | --- | --- | --- |
| **Control** | 18.4 | 20.1 | 9.4 |
|  | 21.6 | 22.9 | 6 |
|  | 19.2 | 21 | 9.7 |
|  | 18.8 | 20.1 | 6.9 |
|  | 20.4 | 22.6 | 10.9 |
|  | 18.2 | 19.5 | 7.3 |
|  | 18.6 | 20.4 | 9.5 |
|  | 19.2 | 22.3 | 16 |
|  | 18.4 | 19.8 | 7.6 |
| **Vehicle + DSS** | 19.9 | 18.3 | -8 |
|  | 20.3 | 20.1 | -1 |
|  | 21.4 | 21.2 | -1 |
|  | 20.3 | 19.5 | -4 |
|  | 19.1 | 19.1 | 0 |
|  | 21.4 | 20.8 | -3 |
|  | 19.8 | 20.2 | 2 |
|  | 19.6 | 19.4 | -1 |
|  | 22.5 | 23.2 | 3 |
| **EcNC101 + DSS** | 20.4 | 19.6 | -4 |
|  | 20 | 19.8 | -1 |
|  | 20.5 | 21.3 | 4 |
|  | 19.1 | 17.6 | -8 |
|  | 19.6 | 18.4 | -6 |
|  | 19.4 | 18.4 | -5 |
|  | 19.1 | 16.4 | -14 |
|  | 20.2 | 17 | -16 |
|  | 19.6 | 16.9 | -14 |

**Supplementary table S2. Raw body weight data of mice that received putrescine supplementation after EcNC101 colonization**

| **Groups** | **Initial body weight (g)** | **Final body weight (g)** | **Body weight variation (%)** |
| --- | --- | --- | --- |
| **EcNC101 + Vehicle + DSS** | 20.5 | 18.1 | -11.7 |
|  | 20.6 | 18.5 | -10.2 |
|  | 19.7 | 17.4 | -11.7 |
|  | 19.2 | 16.9 | -12 |
|  | 22.3 | 21 | -5.8 |
|  | 18.9 | 19.2 | 1.6 |
|  | 18.9 | 19.2 | 1.6 |
|  | 19.7 | 17.4 | -11.7 |
|  | 20.1 | 19.7 | -2 |
|  | 20.4 | 19.3 | -5.4 |
|  | 18.3 | 18.7 | 2.2 |
|  | 18.1 | 18.6 | 2.8 |
|  | 18 | 19.4 | 7.8 |
| **EcNC101 + Putrescine + DSS** | 21.3 | 20.7 | -2.8 |
|  | 22.9 | 23.3 | 1.7 |
|  | 19.3 | 20.4 | 5.7 |
|  | 20.8 | 21.7 | 4.3 |
|  | 19.7 | 20.6 | 4.6 |
|  | 21.1 | 21.1 | 0 |
|  | 21.2 | 22.3 | 5.2 |
|  | 18.2 | 20.7 | 13.7 |
|  | 17.9 | 18.9 | 5.6 |
|  | 20.1 | 22.1 | 10 |
|  | 19.2 | 20.8 | 8.3 |
|  | 19.9 | 20.6 | 3.5 |
|  | 22.6 | 22 | -2.7 |

**Supplementary table S3. Raw body weight data of mice administered oral *B. pseudolongum***

| **Groups** | **Initial body weight (g)** | **Final body weight (g)** | **Body weight variation (%)** |
| --- | --- | --- | --- |
| **EcNC101 + Vehicle + DSS** | 22.6 | 21.3 | -5.8 |
|  | 21.8 | 20.9 | -4.1 |
|  | 20.9 | 20.8 | -0.5 |
|  | 18.8 | 19.9 | 5.9 |
|  | 19.5 | 19 | -2.6 |
|  | 20.7 | 19.7 | -4.8 |
|  | 21.7 | 20.5 | -5.5 |
|  | 20.4 | 19.7 | -3.4 |
|  | 20.6 | 19.1 | -7.3 |
|  | 21.9 | 20.8 | -5 |
|  | 22.1 | 21.5 | -2.7 |
|  | 20 | 19.3 | -3.5 |
| **EcNC101 + *B. pseudolongum +* DSS** | 21.3 | 20.7 | -2.8 |
|  | 20.2 | 21 | 4 |
|  | 22.1 | 21.2 | -4.1 |
|  | 20 | 20 | 0 |
|  | 21.1 | 20.4 | -3.3 |
|  | 20.2 | 20.6 | 2 |
|  | 21 | 22 | 4.8 |
|  | 23.2 | 22.4 | -3.4 |
|  | 21.6 | 22.4 | 3.7 |
|  | 21.5 | 23.1 | 7.4 |
|  | 19.3 | 20.1 | 4.1 |
|  | 22 | 23.6 | 7.3 |
